# Cell-free mitochondrial DNA as a pro-inflammatory agent in blood circulation: mechanisms, therapeutic implications, and clinical challenges in immune dysregulation

**DOI:** 10.3389/fimmu.2025.1640748

**Published:** 2025-10-01

**Authors:** Yangyang Zhao, Chunlei Wu, Xiaoxue Liang, Mengjiao Yang

**Affiliations:** ^1^ Department of Blood Transfusion, Affiliated Hospital of North Sichuan Medical College, Nanchong, Sichuan, China; ^2^ Department of Blood Transfusion, Beijing Anzhen Nanchong Hospital of Capital Medical University & Nanchong Central Hospital, Nanchong, Sichuan, China; ^3^ Department of Medical Laboratory, Chengdu Qingbaijiang District People’s Hospital, Chengdu, Sichuan, China; ^4^ Department of Cardiovascular Surgery, Affiliated Hospital of North Sichuan Medical College, Nanchong, Sichuan, China; ^5^ Graduate School of Comprehensive Human Science, University of Tsukuba, Tsukuba, Japan

**Keywords:** immunity, mitochondrial DNA, cell-free DNA, blood circulation, mitochondria, extracellular vesicles

## Abstract

Circulating cf-mtDNA has emerged as a dual-functional entity in human pathophysiology, serving not only as a disease biomarker but also as a potent innate immune activator through its molecular pattern recognition. Extracellular mtDNA engages PRRs, triggering dysregulated pro-inflammatory signaling in multiple cell lineages. Elevated mtDNA in circulation correlates with pathogenesis of autoimmune disorders, infectious diseases, critical illnesses, neurological disorders, and hematological abnormalities. Therapeutic strategies combining mtDNA monitoring with inhibitors targeting its release mechanisms and downstream pathways offer novel immunomodulatory strategies. This review systematically examines the therapeutic nexus of blood-derived mtDNA in immune activation and disease progression. Here we aim to elucidate the function of mtDNA in disease pathobiology while highlighting mitochondria’s central position in human systemic homeostasis.

## 1 Introduction

Mitochondria serve as the primary sites for energy production in eukaryotic cells and crucial reservoirs for effector molecules regulating fundamental cellular and physiological processes (1, 2). Various components of mitochondria and their metabolic byproducts released from damaged mitochondria exhibit immunogenicity, eliciting immune responses characterized by the presence of damage-associated molecular patterns (DAMPs) (3, 4). Specifically, mitochondrial DAMPs (mtDAMPs) encompass entities such as mitochondrial DNA (mtDNA), cardiolipin, N-formyl peptides (NFP), reactive oxygen species (ROS), adenosine triphosphate (ATP), and mitochondrial transcription factor A (TFAM) (5, 6). Recently, mtDNA has emerged as one of the most extensively researched DAMPs.

Mitochondria evolved from bacterial ancestors and retained a circular chromosome termed mtDNA. In vertebrates, this maternally inherited circular mtDNA is capable of self-replication (1, 7). It encodes 11 subunits of the electron transport chain (ETC) and 2 subunits of ATP synthase, which critical for the oxidative phosphorylation (OXPHOS) (8, 9). Circulating cell-free DNA (cfDNA) has emerged as a novel biomarker with diverse applications across various fields, including oncology, toxicology, cardiovascular diseases (CVD), and organ transplantation (10–14). The primary sources of circulating cfDNA are nuclear DNA (nDNA) and mtDNA (15). Healthy individuals possess approximately 50,000 times more copies of mitochondrial genomes in plasma compared to nuclear genomes, constituting 10% to 25% total circulating cfDNA (16). Circulating mtDNA demonstrates superior immunogenicity compared to Circulating nDNA (17, 18). While optimal mtDNA can maintain mitochondrial genome stability and facilitating its repair mechanisms, supraphysiological concentrations induce cellular damage and trigger immunity (19). As noted by Trumpff et al., the enhanced stability of cf-mtDNA as a biomarker stems from dual protective mechanisms that physical shielding via encapsulation within intact mitochondrial membranes or lipid vesicles, or through protein binding (e.g., TFAM-DNA adduct) (19). And intrinsic resistance to circulating nucleases conferred by its circular double-stranded topology and lack of histone association (20).

mtDNA exhibits a dual role as a tissue damage biomarker and DAMP, activating innate immunity via pattern recognition receptors (PRRs). Current research focuses on enhancing our understanding of the regulation of mtDNA in circulation and its influence on disease severity. A critical gap remains in pleiotropic injury mechanisms of systematizing mtDNA. Nevertheless, a comprehensive review of the mechanisms through which mtDNA induces damage have yet to be thoroughly examined.

## 2 Circulating mtDNA: from intracellular transport to extracellular release

### 2.1 mtDNA is released from mitochondria into the cytoplasm

**Figure 1:**
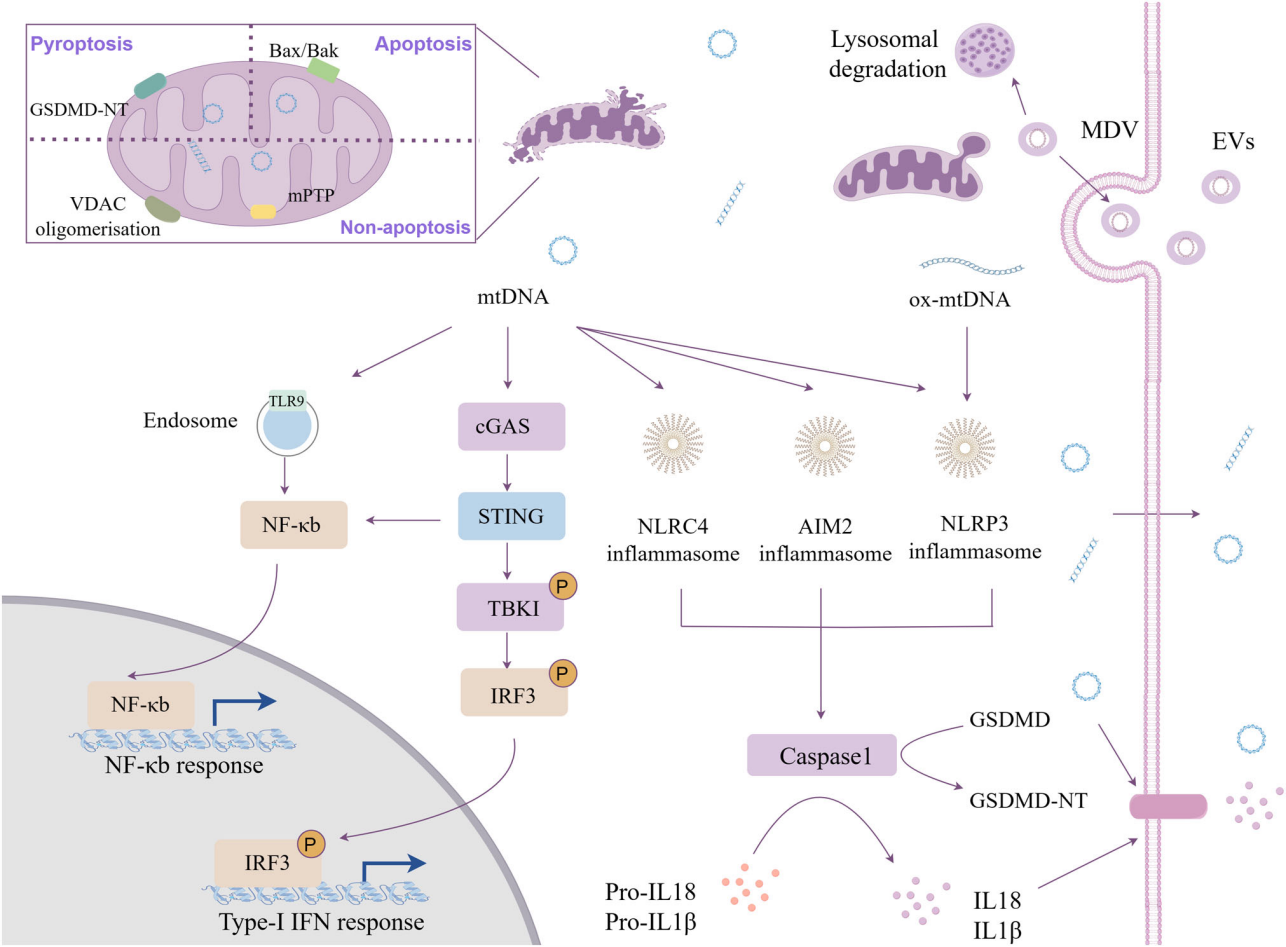
Cytoplasmic mtDNA mediated innate inflammation. The release of mtDNA into the cytoplasm occurs through the mitochondrial inner and outer membrane permeability transition pore, activating multiple pattern recognition receptors. Besides, mtDNA be packaged into mitochondrial-derived vesicles and transferred to recipient cells through extracellular vesicle mechanism.

Structurally, mitochondrial compartmentalization relies on its dual-membrane architecture—an inner mitochondrial membrane (IMM) and an outer mitochondrial membrane (OMM)—that sequester mtDAMPs from cytosolic PRRs (21). mtDNA release from the mitochondrial matrix into the cytosol must traverse both membranes (22) (**Figure 1**). Current researches indicate several major pathways regulate in the permeability of mitochondrial membranes, including the mitochondrial permeability transition pore (mPTP), Bcl-2-associated X protein (BAX)/Bcl-2 homologous antagonist/killer (BAK), voltage-dependent anion channel (VDAC), and gasdermin D (GSDMD). Activation of these channels induces mitochondrial depolarization, disruption of OXPHOS system, mitochondrial membrane permeabilization, and mtDNA extrusion (23–25). BAX and BAK are proapoptotic pore-forming proteins that mediate mitochondrial outer membrane permeabilization (MOMP), a process enabling release of cytochrome c and mtDNA to trigger apoptosis while impairing mitochondrial respiration (5, 25). The VDAC, a β-barrel membrane protein located at the OMM, involves the exchange of materials between mitochondria and the cytoplasm, maintains intracellular calcium homeostasis, and regulates apoptosis and necrosis (26, 27). During pyroptosis, caspase-mediated cleavage generates GSDMD-N-terminal fragments (GSDMD-NT) that target cardiolipin-containing mitochondrial membranes. This interaction disrupts mitochondrial phospholipid bilayers, facilitating mtDAMPs release prior to plasma membrane rupture in a cell lysis-independent manner (23, 28, 29). Furthermore, pyroptosis disturbs mitochondrial homeostasis and induces MOMP through membrane depolarization, ionic imbalance, and suppressed mitophagy (5). The mPTP, a non-specific channel located in the IMM, activates under stress conditions including Ca2^+^ influx, oxidative stress, BAX/BAK oligomerization, and VDAC (25, 30, 31) (**Figure 1**).

### 2.2 Cytosolic mtDNA engages multiple pattern recognition receptors

Augmented immunogenicity of mtDNA attributable to its unique molecular architecture: high copy number, circular hypomethylated structure, and lacking free DNA termini (2, 29, 32). Simultaneously, these characteristics render mtDNA readily identifiable and capable of binding, which can elicit substantial biological effects. It is recognized by several PRRs: cyclic guanosine monophosphate adenosine monophosphate (cGAMP) synthase (cGAS), nucleotide oligomerization domain (NOD)-like receptors (NLRs), and Toll-like receptor 9 (TLR9) (33–35) (**Figure 1**).

**cGAS:** cGAS functions as a crucial cytosolic DNA sensor, activated in sequence-agnostic through hybridization with double-stranded DNA (dsDNA) (36). cGAS-mtDNA binding catalyzes the synthesis of the second messenger cyclic GMP-AMP (cGAMP) (37). This molecule subsequently regulates the downstream stimulator of interferon genes (STING) pathway, wherein STING1 activation ultimately recruits TANK-binding kinase 1 (TBK1) (37, 38). Then, TBK1 catalyzes the phosphorylation of interferon regulatory factor 3 (IRF3) and promotes nuclear factor kappa-B (NF-κB) signaling, leading to the production of type I interferons (IFNs) and other pro-inflammatory cytokines (37–39).

**Inflammasomes:** Beyond cGAS-mediated pathways, cytosolic mtDNA engages absent in melanoma 2 (AIM2) via C-terminal hematopoietic interferon-inducible nuclear (HIN) domain and NLRP3/NLRC4 inflammasomes, facilitating the secretion of proinflammatory cytokines (29, 40, 41).

**TLR9**: mtDNA retains evolutionarily conserved CpG motifs resembling bacterial nucleic acids, serving as potent TLR9 agonists through their hypomethylated DNA architecture (42). Cytosolic mtDNA containing CpG motifs will stimulate endolysosomal TLR-9 to recruit myeloid differentiation primary response 88 (MyD88), activating transcription factors such as NF-κB, driving pro-inflammatory cytokine and chemokine cascades (43, 44).

Transcriptional factor a mitochondrial (TFAM)-bound mtDNA resists ROS-mediated oxidation through its compact nucleoid structure within mitochondria (45, 46). In contrast, newly synthesized naked mtDNA, lacking TFAM shielding and other protective mechanisms, is prone to oxidative modification by OXPHOS-derived mtROS due to spatial proximity to the OXPHOS (30, 47). This susceptibility can result in the leakage of mtDNA into the cytoplasm (48, 49). Unlike nuclear DNA, mtDNA lacks histone protection and effective DNA repair mechanisms, rendering it more vulnerable to damage (50). The oxidized form of mtDNA (ox-mtDNA) acts as a potent DAMP that drives sustained pattern recognition receptor activation and amplifies sterile inflammation (30). However, the molecular mechanisms underlying mtDNA oxidation and fragmentation under various stress conditions require further investigation. Subsequent research demonstrates that ox-mtDNA released into the cytosol during mitochondrial dysfunction serves as an effective activator of the NLRP3 inflammasome and TLR9 (51, 52). The NLRP3 inflammasome appears to preferentially respond to oxidized DNA, while AIM2 is proposed to primarily recognize non-oxidized DNA (24, 47). Topoisomerase deficiency, which serve as genetic and pharmacological triggers of mitochondrial genome instability, induces left-handed Z-form mtDNA accumulation (53). This form of mtDNA is more readily recognized by the nucleic acid sensor Z-DNA binding protein 1 (ZBP1) (53). Recent studies reveal ZBP1 coordinates with cGAS and receptor-interacting protein kinase 1/3 (RIPK1/3) to sustain the type I interferon (IFN) signaling pathway activated by mtDNA instability (53, 54). Cytosolic DNA sensors differentiate mtDNA types. How distinct mtDNA forms engage specific PRRs to drive unique inflammatory responses remains unclear.

### 2.3 mtDNA is released from the cytoplasm to the extracellular milieu

Under pathological conditions, mtDNA escapes into the cytosol and extracellular space through three primary pathways: 1) regulated cell death (RCD): cfDNA is released into the circulation after apoptosis, necroptosis, and pyroptosis (28, 55, 56). Both necroptosis and pyroptosis result in the rupture of the cytoplasmic membrane; necroptosis is mediated by the oligomerization of mixed lineage kinase domain-like proteins (MLKLs), while pyroptosis is mediated by GSDMD (28, 56). Mitochondrial permeability transition (MPT)-driven necrosis is a necrotic variant of regulated cell death that can eventually result in the complete disintegration of mitochondrial membranes (57). This process is characterized by a rapid depletion of ATP and oxidative damage to macromolecules, occurring independently of caspase activation (58); 2) the efflux of mtDAMPs due to defects in mitochondrial quality control (MQC) (59); and 3) the active secretion of mitochondrial-derived vesicles (MDVs) (55, 60). Additionally, the leakage of mtDNA into the cytosol, whether in circular or fragmented form, can result from mitochondrial damage caused by oxidative stress and damage to membrane structures (61). The accumulation of mtDNA in the cytosol activates intracellular inflammatory signaling pathways, which subsequently alter the physiological state of cells and cause cell death (60). This process further contributes to the release of mtDNA into the extracellular environment. Mitophagy as part of the MQC could inhibit mtDNA leakage by facilitating the disposal of dysfunctional mitochondria and limit potential pro-inflammatory effect (60). Following the activation of PTEN-induced putative kinase 1 (PINK1)- and parkin (PRKN)-mediated mitophagy, the ubiquitination of mitochondrial proteins that induce oxidative damage takes place. Subsequently, these ubiquitinated mitochondria are engulfed by autophagosomes, which then coalesce with lysosomes to facilitate the systematic degradation and recycling of mitochondrial components (62). Sublethal MOMP activates mitophagy, enabling the processing of dysfunctional mitochondria through lysosomal degradation (24, 63).

### 2.4 Multiple forms of cf-mtDNA

**Figure 2:**
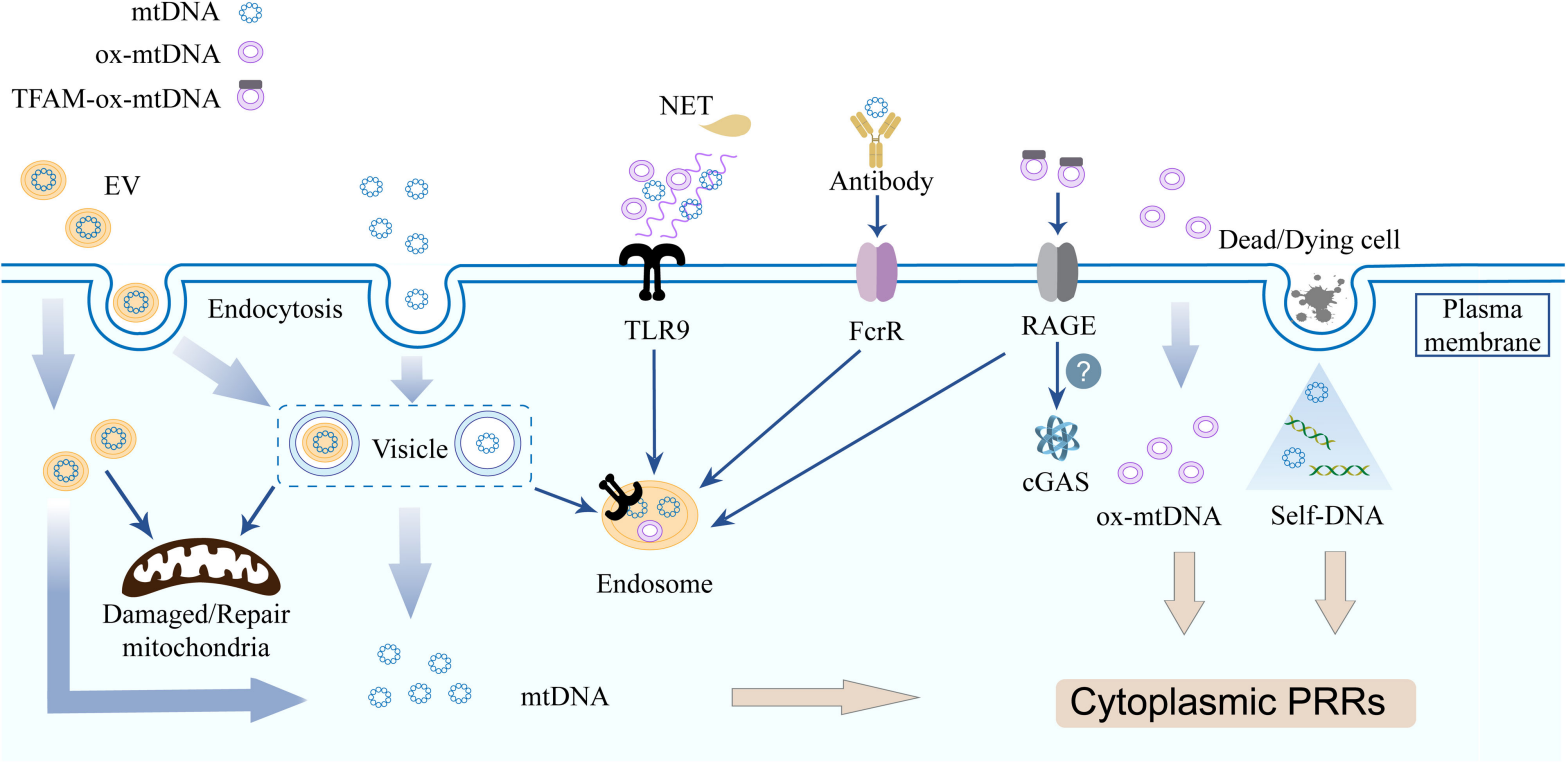
Cell free mitochondrial nucleic acids triggers inflammation via multiple distinct pathways. Innate immune cells acquire cf-mtDNA via endocytosis (EVs/free forms) or phagocytosis. mtDNA from deading/dying cells released during infection bind PRRs. TLR9 recognizes NET-DNA, mtDNA, and ox-mtDNA. TFAM-associated ox-mtDNA internalized by pDC via RAGE receptors, the IFN-generating sensor in recipient cells is unclear.

Current evidence indicates that circulating cf-mtDNA exists in heterogeneous forms. These range from distinct compositional states, including naked DNA, lipid vesicle-encapsulated DNA, intact mitochondria, and neutrophil extracellular traps (NETs), to different modes of circulation, such as free diffusion or vesicle- mediated transport (**Figure 2**).

Naked as well as protein-bound mtDNA molecules are released into the extracellular microenvironment, where they functions as an autocrine, paracrine, or endocrine immune stimulus (24). TLR9 is primarily expressed in innate immune cells, B lymphocytes, and certain non-immune cell lineages. Both mtDNA and bacterial DNA possess low-methylated CpG dinucleotide motifs that can be recognized by TLR9 (64). In addition to being recognized directly by TLR9 on the cell membrane, extracellular mtDNA activates TLR9 occurring via two principal routes: phagocytosis of mtDNA-containing cellular debris or cells, and internalized through autophagic delivery of mitochondrial fragments within the endolysosomal compartment (65–67). Furthermore, the dissemination of mtDNA may occur through direct fusion between the mitochondrial membrane and the cell membrane; this fusion event has been associated with elevated levels of ox-mtDNA (51, 68).

Extracellular vesicles (EVs) represent a significant source of cf-mtDNA (69). EVs are lipid bilayer membrane vesicles found in body fluids, essential for intercellular communication, maintaining internal balance, and promoting pathological processes (70, 71). EVs, as crucial transport carriers for mtDNA and play a significant role in the sorting of mitochondrial components via the dual MDVs. The Snx9-dependent MDVs facilitates the transport of intact IMM and matrix proteins to EVs, whereas the Parkin-dependent MDVs is responsible for targeting oxidatively damaged mitochondrial components for lysosomal degradation (62). Generally, EVs contain original cell membrane structures and cytoplasmic components, playing a crucial role in intercellular signaling (72, 73). EVs have the capacity to transfer mtDNA to another cells, modifying the mitochondrial function or metabolic state of the recipient cells. This mechanism may be involved in tissue repair, immune regulation, and various pathological processes. Innate immune cells can uptake either EVs or cell free nucleotide through endocytosis, as well as mtDNA from cells and cell debris through phagocytosis (29, 74). TLR9 may detect EVs containing mtDNA (mtDNA-EVs) through endolysosomal compartments in a cell-autonomous manner (29). Specifically, PINK1 plays a crucial role in the packaging of mtDNA into EVs through a scaffolding mechanism that is independent of its kinase activity and traditional mitophagy processes. Furthermore, PINK1 can promote the release of mtDNA-containing EVs in breast cancer cells. This process results in the autocrine and paracrine activation of TLR9, which subsequently enhances the degradation of the extracellular matrix and the invasive capabilities of the cancer cells (75). Activated T cells release mtDNA-EVs, which stimulate the cGAS/STING pathway in dendritic cells (76). In a distinct clinical context, EVs have garnered significant attention across various domains, including drug delivery and regenerative medicine, owing to their numerous advantages such as high biocompatibility, low cytotoxicity, low immunogenicity, and a rich content of growth factors (77, 78). Mitochondrial transplantation via EVs ameliorates dysfunction in Leigh syndrome models (79). The nanoscale dimensions of EVs present significant challenges for current analytical systems in verifying or regulating their effects. This situation underscores the need for more advanced analytical techniques capable of detecting EV subpopulations of varying sizes, which would enhance the sensitivity and specificity of EV detection. Furthermore, critical unknowns include EV-delivered mtDNA integration/expression in recipient cells and its signaling mechanisms.

NETs are DNA mesh structures that function to capture and eliminate pathogens; however, excessive formation of these structures contributes to diverse pathologies (80). Initially, it was widely believed that NETs were primarily composed of nDNA, emerging evidence demonstrates the presence of mtDNA within NETs under specific stimulating conditions (81). Neutrophils release mtDNA through a process known as NETosis, and mtDNA can trigger the formation of NETs (15, 81, 82). The NETosis described by Yousefi et al. revealed that mtDNA that is released rather than nDNA during this process (81). Beyond active extrusion via NETosis, extracellular mtDNA accumulation may also passive diffusion from other leukocytes and necrotic cells (83, 84). Accumulating evidence links dysregulated NETosis to multiple inflammatory and autoimmune conditions, including atherosclerosis, psoriasis, rheumatoid arthritis (RA), gout, anti-neutrophil cytoplasmic antibody-associated vasculitis, and systemic lupus erythematosus (SLE) (85, 86). Characteristically, NET-derived DNA complexes amplify TLR7/9 signaling in plasmacytoid dendritic cells (pDCs) or pancreatic ductal epithelial cells, driving cytokine production. These cytokines reciprocally enhance NETosis, establishing a self-perpetuating inflammatory loop (87, 88).

The diagnostic research concerning mtDNA encounters numerous challenges and issues. A significant concern is the absence of a comprehensive classification system to differentiate between extracellular mtDNA (ex-mtDNA) and various biological forms of intact mitochondria (89). Caicedo et al. have delineated four categories of ex-mtDNA (89). These distinct forms of ex-mtDNA demonstrate varying levels and configurations under different health and disease states. Factors such as sample type, collection methods, processing techniques (including anticoagulant selection and centrifugation parameters), and storage conditions significantly influence detection accuracy, particularly in relation to the prevention of false positives resulting from platelet activation (16, 90). Therefore, the establishment of a standardized classification system and detection protocol is essential for enhancing the clinical diagnostic utility of mtDNA.

## 3 The effect of cf-mtDNA in blood circulation

### 3.1 Autoimmunity

Autoimmune diseases represent a complex array of disorders driven by intricate interactions between genetic predisposition and environmental triggers. Recent studies have increasingly highlighted the pathogenic significance of mtDNA in facilitating autoimmune diseases. Under physiological conditions, circulating cf-mtDNA is regulated by the activity of DNase, which maintains low levels to prevent aberrant immune activation. Clinically, impaired DNase activity has been implicated in the pathogenesis of multiple autoimmune disorders, highlighting its potential protective function (91). Mutations in the mitochondrial genome or prolonged exposure to pro-inflammatory cytokines promote mitochondrial dysfunction, leading to the release of mitochondrial components that initiate innate immune activation (92). Furthermore, the presence of antibodies targeting mitochondrial nucleic acids (mtDNA and mitochondrial RNA) in autoimmune diseases demonstrats active cross-talk between mitochondrial constituents and adaptive immunity (93–95). Pathological amplification of type I IFN response via self-DNA (including mtDNA) sensing underlies severe autoinflammatory manifestations, as observed in Aicardi-Goutières syndrome and infantile STING-associated vasculopathy (96, 97).

**SLE:** SLE is an autoimmune disorder characterized by high IFN-mediated multisystemic damage, with “DNA overload” emerging as a pivotal pathogenic driver (98, 99). mtDNA functions as a potent type I IFN pathway agonist to facilitate disease progression. Clinically, elevated serum anti-mtDNA IgG antibodies correlate with disease activity and predict nephritis (93). Plasma cf-mtDNA predominantly originates from naked mitochondria, with platelets serving as a major source (100). In SLE patients, the release of mtDNA is linked to platelet degranulation mediated by platelet FcγRIIA and the fibrinogen receptor α2bβ3 (101). When hydrolyzed by secretory phospholipase A2 group IIA, naked mitochondria release pro-inflammatory lipid mediators and mtDNA to enhance neutrophil activation (102). Furthermore, GSDMD not only facilitates the release of ex-DNA but requires ox-mtDNA-mediated GSDMD-N oligomerization, establishing a self-reinforcing cycle of lytic cell death that promotes the release of extracellular DNA and pro-inflammatory PCD in neutrophils in SLE (103). TLR9 and RAGE are involved in the uptake of extracellular TFAM-associated ox-mtDNA nucleoids by pDCs, thereby stimulating IFN production (51)(**Figure 2**). The pharmacological inhibition of VDAC-mediated mtDNA release, as well as the application of the mtROS scavenger MitoTEMPO, has been shown to reduce disease severity in murine models of lupus (68, 104).

**RA:** Elevated circulating and synovial cf-mtDNA in RA patients correlates with disease activity and serves as an early diagnostic biomarker (11, 91, 105). Crucially, mtDNA drives RA pathogenesis through multiple pro-inflammatory mechanisms: Platelet-derived microparticles carrying mitochondria contribute to immune complex formation and stimulate monocytes to release IL-1β and TNF-α (106, 107); mtDNA-exposed synovial neutrophils upregulate receptor activator of nuclear factor kappa-B ligand, promoting joint erosion (108). Supporting its pathogenic centrality, impaired mtDNA clearance triggers RA-like arthritis in mice (17, 109), anti-inflammatory therapies reduce cfDNA (91), and TLR9 inhibition hydroxychloroquine (HCQ) shows clinical efficacy (110). Expanding beyond canonical pathways of lysosomal pH elevation, TLR signaling inhibition, and cytokine regulation, HCQ suppresses TLR-mediated inflammation via the RNF13-LAMP-1 axis (111, 112). Additionally, racemic HCQ inhibits fibroblast-like synoviocyte function by blocking the PI3K/AKT pathway, thereby ameliorating synovitis (113). These findings collectively demonstrate the capacity of HCQ to modulate RA pathogenesis through multitargeted synergistic mechanisms.

**Systemic sclerosis (SSc)**: In SSc, elevated plasma mtDNA levels correlate with TLR9 and cGAS pathway stimulation, inducing pathogenic IFN and IL-6 production that parallels declines in lung function (forced vital capacity) (114, 115). Critically, the increased of mtROS is driven by the inhibition of PINK1/Parkin-mediated mitophagy in type II alveolar epithelial cells, causing ox-mtDNA damage and suppressing DNA repair mechanisms (116).

### 3.2 Neurological disorders

In the field of neurological disorders, circulating cf-mtDNA serves as a potential biomarker for mitochondrial damage, neuroinflammation, and stress responses, exhibiting abnormal concentrations in multiple conditions including Parkinson’s disease (PD), Alzheimer’s disease (AD), multiple sclerosis (MS), and bipolar disorder (BD). However, significant inconsistencies exist across studies due to variables such as sample source (peripheral blood vs. cerebrospinal fluid), disease subtype heterogeneity, and methodological differences in detection (117–119). This issue has been elaborated in detail in the systematic review by Risi et al. (120).

cf-mtDNA not only correlates with neuroinflammatory progression in depression, dementia, and amyotrophic lateral sclerosis (ALS) but also directly contributes to pathogenesis through specific molecular mechanisms (121–123). Notably, current research on the pathogenic mechanisms of cf-mtDNA remains limited, with studies predominantly concentrated on its biomarker utility.

Serum cf-mtDNA was significantly elevated in ALS, particularly in SOD1 mutation carriers, and positively correlate with IL-6 levels and disease progression rate, indicating synergistic roles of mitochondrial dysfunction and neuroinflammation in pathogenesis (122). In narcolepsy type 1, elevated cerebrospinal fluid cf-mtDNA inversely correlates with hypocretin-1 concentration and associates with sleep architecture abnormalities. Concurrent changes in IL-6/IL-18 further implicate neuroinflammation in disease pathology (124). In BD, cf-mtDNA positively correlates with C-reactive protein (CRP), suggesting involvement in disease progression via inflammatory pathway activation and interaction with metabolic syndrome-associated low-grade inflammation (125). Together these studies suggest a potential link but not causality between cf-mtDNA and neuroinflammation pathologies.

Mechanistically, Tripathi et al. demonstrated that chronic restraint stress significantly increases serum cf-mtDNA in mice. They established the centrality of the cf-mtDNA-TLR9 signaling axis in mediating social behavior deficits, triggering neuroinflammation in the prefrontal cortex and ultimately driving behavioral abnormalities (126). In the chronic intermittent ethanol exposure mouse model, high numbers of mtDNA-EVs could promote in disease progression, exacerbating neuroinflammation and compromising the integrity of the blood-brain barrier (127). It’s also worth mentioning that the cGAS-STING pathway activated by mtDNA plays dual roles in neurodegeneration: while transient activation confers neuroprotection, excessive or chronic CNS stimulation drives neuroinflammation and neurodegeneration (43).

### 3.3 Infectious diseases and critical illnesses

Circulating cf-mtDNA has been consistently associated with the onset, severity, and prognosis of diverse diseases across multiple studies, demonstrating significant diagnostic potential in infectious diseases and critical illnesses. Concurrently, the inflammatory roles of cf-mtDNA in disease pathogenesis are actively being elucidated. Under pathogenic stimuli such as viruses or bacteria, mtDNA can be released from damaged mitochondria into the cytoplasm and subsequently enter the systemic circulation via active or passive release mechanisms (128, 129). In chronic inflammatory diseases, persistently elevated circulating cf-mtDNA is linked to progressive cellular stress and death (22, 59). During acute disease or injury, a marked increase in circulating cf-mtDNA can trigger acute systemic inflammatory response syndrome (SIRS) (130).

**Cardiac dysfunction**: Circulating cf-mtDNA levels correlate significantly with 30-day mortality in cardiogenic shock and decompensated heart failure patients, but lack prognostic value in cardiac arrest (where uric acid demonstrates superior predictive utility). This disease-specific association positions mtDNA as a targeted prognostic biomarker for cardiac dysfunction-related critical illness (131).

**Severe fever with thrombocytopenia syndrome (SFTS):** SFTS is an infectious disease caused by the tick-borne SFTS virus, with a high case fatality rate ranging from 10% to 50%. Studies have demonstrated significantly elevated circulating cf-mtDNA in SFTS patients, which strongly correlate with adverse clinical outcomes. Mechanistically, endothelial cell-derived mtDNA promotes B-cell activation, migration, and differentiation via the TLR9 pathway, enhancing B-cell susceptibility to SFTSV infection, thereby facilitating viral replication and exacerbating disease progression (132).

**COVID-19:** Circulating cf-mtDNA levels distinguish COVID-19 clinical subtypes (133–135). Critical cases: Non-survivors exhibit 76% lower mtDNA abundance and shorter fragments vs. survivors; elevated cf-mtDNA correlates with ICU admission/death risk (positive predictive value for mortality: 83.3%). Long COVID: Reduced cf-mtDNA with mitochondrial structural abnormalities indicate persistent mitochondrial dysfunction. Asymptomatic individuals show higher cf-mtDNA than symptomatic patients, who conversely exhibit elevated cf-nDNA (135, 136). During respiratory failure, serum cf-mtDNA levels are elevated and positively correlate with oxygen therapy requirement (137). In COVID-19-associated myocarditis models, cf-mtDNA activates TLR/NF-κB signaling, exacerbating myocardial injury via pro-inflammatory cytokine release in myocarditis models (128).

**Sepsis**: Sepsis, a life-threatening systemic inflammatory response to infection, manifests as multiorgan dysfunction with high mortality rates (138). circulating cf-mtDNA from damaged tissues, which functions as a DAMP to hyperactivate innate immunity, intensifying systemic inflammation and impairing organ function during the progression of sepsis (139). both the administration of mitochondrial fragments enriched with mtDNA in murine models and *in vitro* and *in vivo* mtDNA injections can induce comparable inflammatory cascade observed in clinical sepsis (139, 140). Clinical investigations demonstrate that circulating cf-mtDNA increased markedly in sepsis patients correlate with the onset of acute kidney injury (AKI), acute lung injury (ALI), and acute respiratory distress syndrome (ARDS) (139, 141, 142). In sepsis-induced AKI, mtDNA can enhance mitochondrial oxidative stress driving a self-reinforcing pathological loop (139). Clinical validation studies demonstrate strong correlations between circulating cf-mtDNA levels and AKI severity markers. Depletion of mtDNA mitigates acute tubular cell injury, indicating that position mtDNA-centric therapeutics as promising investigational approaches for AKI (143). Notably, hemodialysis patients with elevated circulating cf-mtDNA exhibit higher risks of adverse clinical outcomes (144). In the proinflammatory microenvironment in end-stage renal disease, the immunogenicity of circulating cf-mtDNA may adversely affect patient health, though lacking all-cause mortality association (145). In sepsis-associated ALI and ARDS, circulating cf-mtDNA activates a robust STING pathway in macrophages, disrupting autophagic flux by impairing lysosomal acidification in an IFN-dependent manner, thereby exacerbating lung endothelial barrier disruption and propagate a cytokine storm (141).

### 3.4 CVD

The concentration, copy number, and methylation profiles of circulating cf-mtDNA serve as critical indicators of disease status, progression stage, and prognostic risk, demonstrating significant clinical potential across cardiovascular and related disorders including diabetic macroangiopathy, abdominal aortic aneurysm (AAA), and atrial fibrillation (AF) (146–149). Additionally, cf-mtDNA abnormalities are closely linked to mitochondrial functional decline and accelerated biological aging: In chronic kidney disease (CKD), low mtDNA copy number (mtDNA-cn) coupled with high cf-mtDNA levels associates significantly with vascular calcification and epigenetic age acceleration (150).

Among heart failure (HF) and type 2 diabetes mellitus (T2DM) patients, heightened cf-mtDNA correlates with mitochondrial dysfunction and metabolic stress, exhibiting positive associations with systemic inflammatory markers—indicating its role as a mediator of metabolic-inflammatory crosstalk in disease progression (151). Individuals diagnosed with T2DM exhibit elevated circulating cf-mtDNA, driven by chronic hyperglycemia-induced mtROS and mitochondrial dysfunction (152, 153). A weak correlation has been identified between plasma IL-1β levels and circulating cf-mtDNA. Mechanically, circulating cf-mtDNA activates the AIM2 inflammasome in macrophages, triggering caspase-1-dependent IL-1β/IL-18 maturation and secretion (152). Additionally, cerebral vessel remodeling and impaired cerebrovascular reactivity may be associated with variations of mtDNA and inflammation particularly in early diabetic kidney disease, yet the precise mechanisms underlying mtDNA-driven cerebrovascular remodeling remain incompletely characterized (154). Mitochondrial debris from tubular and glomerular cells enters systemic circulation in diabetic kidney disease (155). In blood and urine, mtDNA levels were evaluated as a specific signature in relation to inflammatory response within the diabetic kidney at the glomerular and tubular (156). In maintenance hemodialysis (MHD) patients, exogenous cf-mtDNA upregulates TLR9, ICAM-1 and TNF-α in cardiac microvascular endothelial cells, intensifying microvascular inflammation and CVD progression (157).

**AAA**: In AAA, cf-mtDNA derived from patient peripheral blood mononuclear cells (PBMCs) stimulates macrophages to potentiate AIM2/IFI16 inflammasome assembly, upregulating apoptosis-associated speck-like protein (ASC) and IL-1β expression, thereby inducing ASC speck formation and exacerbating chronic aortic wall inflammation (146).

**Myocardial ischemia/reperfusion (MI/R)**: During MI/R injury, cf-mtDNA activates the NLRP3 inflammasome in a TLR9-dependent manner, mediating splenic monocyte inflammatory responses that amplify IL-1β release and augment infarct size (158).

**Metabolic complications with CVD**: Patients with metabolic syndrome (MetS) exhibit elevated circulating ox-mtDNA and upregulated TLR9 expression in peripheral blood mononuclear cells. *In vitro* stimulation of THP-1 monocytes with cfDNA or ox-mtDNA activates TLR9/NF-κB signaling, driving proinflammatory cytokine secretion in MetS-associated cardiovascular disease (159). Obesity induces elevation of cf-mtDNA in cerebrospinal fluid, particularly found in major target organs in hypertension, such as the heart, kidneys, and brain (160, 161). Mechanistically, cf-mtDNA in the cerebrospinal fluid activates the sympathetic nervous system to cause hypertension via the TGFβ signaling pathway. This neuroimmune crosstalk increases sympathetic output to the cardiovascular system (160).

In summary, while substantial progress has been made in elucidating the roles and mechanisms of cf-mtDNA in CVD, its clinical utility requires further validation through large-scale prospective studies.

### 3.5 blood system diseases

**Sickle cell disease (SCD):** SCD exhibits pathologically elevated circulating cf-mtDNA levels, driven by abnormal mitochondrial retention in erythrocytes and hemolysis-mediated release during vaso-occlusive crises (VOC) (82). Hypomethylated mtDNA triggers the formation of NETs, exacerbating chronic inflammation and organ damage during VOC (82). The retention of functional mitochondria in mature erythrocytes accelerates cellular senescence, driving membrane fragility and hemolysis (162, 163). Accelerated intravascular lysis of sickle RBCs facilitates mitochondrial extrusion into circulation (82). Hemolysis-derived mtDNA can be transferred to antigen-presenting cells (APCs) by other RBCs, engaging TLR9 to potentiate inflammatory cytokines secretion. In SCD, mitochondria-positive RBCs exhibit reduced circulatory half-life, rendering them susceptible to phagocytosis (164). Under physiological conditions, aging or damaged erythrocytes undergo cleared by splenic macrophages without eliciting an immune response; however, in SCD, immunogenic APC subpopulations more effectively facilitate the removal of aging RBCs increasing the possibility of producing autoantibodies (165, 166). Consequently, circulating cf-mtDNA as a byproduct of hemolysis has the potential to activate coagulation and inflammatory pathways. This activation may contribute to self-perpetuating cycle of VOC and end-organ damage (82).

**Multiple myeloma (MM)**: Patients with MM exhibit significantly elevated cf-mtDNA originating from malignant plasma cells in both peripheral blood and bone marrow. These mtDAMPs promote MM progression by activating the STING pathway in bone marrow macrophages, inducing chemokines release and enhancing the retention of MM cells within the bone marrow niche (167).

**Anemia:** RBCs bind and eliminate circulating cf-mtDNA via surface TLR9 receptors in the blood, which subsequently stimulate macrophages to phagocytize these complexes and provoke inflammatory responses (65, 168, 169). This clearance mechanism partially alleviates pulmonary inflammation in ARDS and sepsis patients (65, 169). Nonetheless, pathologically amplified RBC-mtDNA interactions accelerate erythrocyte clearance, contributing to anemia in sepsis, COVID-19, and hematologic malignancies (65, 170, 171).

As a potent proinflammatory mediator, mtDNA provides critical insights into inflammatory disease pathogenesis (**Table 1**). Its dual role as both a DAMP and a biomarker bridges mitochondrial dysfunction with systemic inflammation. Nevertheless, significant gaps remain in our understanding of the specific mechanisms by which circulating cf-mtDNA operates in different diseases. This includes the regulatory mechanisms governing mtDNA release, its interactions with other cellular signaling pathways, and the development of precise therapeutic strategies aimed at targeting circulating cf-mtDNA.

**Table 1:** Prominent examples of pathologies directly linked to circulating cf-mtDNA.

Disease name	Experimental model	Targets	Reference
SLE	pDC	TLR9	(51)
Chronic stress	Mice	TLR9	(126)
SFTS	B cell	TLR9	(132)
SARS-CoV-2–induced acute myocarditis	PBMC	TLR-NF-κB	(128)
sepsis-related acute lung injury	C57BL/6 mice	STING	(141)
AAA	THP-1 cell	AIM2 inflammasome	(146)
MI/R	Mice	TLR9- NLRP3 inflammasome	(158)
CVDs in MetS	THP-1 cell	TLR9-NF-κB	(159)
Obesity-related hypertension	Mice	TGFβ	(160)
MM	Mice	STING	(167)

SLE, systemic lupus erythematosus; pDC, plasmacytoid dendritic cell; SFTS, severe fever with thrombocytopenia syndrome; AAA, abdominal aortic aneurysm; MI/R, myocardial ischemia/reperfusion; CVDs, cardiovascular diseases; MetS, metabolic syndrome; MM, multiple myeloma.

## 4 Pharmacology

mtDNA-driven immunity dysregulation constitutes a pathogenic axis in diverse human diseases, spanning hyperinflammatory conditions to inefficient inflammatory states (35). Pharmacological agents that target mitochondrial function have the potential to modulate inflammatory processes, offering novel therapeutic strategies for mtDNA- driven inflammatory diseases, particularly in scenarios where conventional treatments exhibit limited efficacy. The mitochondrial uncoupler BAM15 can reduce mortality and mitigate kidney injury in sepsis models by breaking the mtDNA-TLR9 feedforward loop that amplifies tissue injury (139).

**Mitochondrial Pore Opening Inhibitors:** Targeted mitochondrial membrane stability emerges as a strategic intervention to prevent pathological mtDNA release. Pharmacological agents such as Cyclosporine A (a Cyclosporine D inhibitor), VBIT-4 (a VDAC inhibitor), and Venetoclax (a BCL-2 inhibitor) is anticipated to facilitate the targeted inhibition of pore opening in the IMM or OMM limiting the release of mtDNA (172–174). Notably, Cyclosporine A is approved for use in humans, primarily for the treatment of autoimmune diseases and the prevention of transplant rejection (58). The application of this is mainly based on its interaction with the cytoplasmic protein PPIF, which inhibits calcineurin and subsequently suppresses lymphocyte activity. Yet the partial immunosuppressive effects of Cyclosporine A through MPT inhibition and the attenuation of mitochondrial-driven inflammatory responses, a hypothesis that warrants further investigation (24). VBIT-4 alleviates symptoms resembling SLE in lupus-prone murine models (104). Some antioxidants sustains mitochondrial function by preserving mitochondrial membrane integrity and reducing pathological mtROS (175).

**Cell Death and Autophagy:** The impairment of mitochondrial function and structural integrity associated with RCD drives pathogenic mtDNA release. Disulfiram blocks the formation of GSDMD pores, reducing ox-mtDNA release and alleviating symptoms in lupus models (103). Hypocrellin A, a component in ethnic medicinal fungus, targets NLRP3 NACHT domain to inhibit the assembly and activation of the inflammasome (176). Mitophagy facilitates the degradation of dysfunctional mitochondria via lysosomal pathways, limiting the release of mtDNA and mtROS and inhibiting PRR signaling and subsequent inflammatory responses (177, 178). Parkin-dependent mitophagy generates mitochondria-derived vesicles from mtDAMP, suppressing paracrine inflammation (179). At present, the specific pharmacological modulators of mitophagy that are available for clinical use remain unclear.

**Targeted Therapies for PRRs and Their Signaling Pathways:** Precision modulation of PRR signaling cascades activated by mtDNA emerges as a strategic frontier in autoimmune therapeutics. Notably, the cGAS-STING signaling pathway has garnered considerable attention, with several inhibitors already available, including the competitive inhibitor of IRF3 activation, known as MITA/STING activation, tetrahydroisoquinoline, and the cGAS cyclic peptide inhibitor XQ2B (180, 181). Hydroxychloroquine, functioning as a TLR-9 inhibitor, has demonstrated efficacy in the treatment of RA, SLE, and various other connective tissue disorders (110, 182). Over the past two decades, monoclonal antibodies (mAbs) targeting type I IFN pathway have undergone extensive evaluation in clinical trials. Among these, belimumab and anifrolumab have received clinical approval for the treatment of SLE (98). Nevertheless, > 50% of patients remain refractory to achieve the anticipated outcomes of disease improvement and reduced flare-ups when treated with these two mAbs, highlighting unmet needs for more effective therapeutic options (98).

Targeting the mtDNA inflammatory pathway offers therapeutic promise for refractory inflammatory diseases, yet several mechanisms and safety concerns must be addressed. Mechanistic ambiguities in drug pharmacology, such as the potential dual immunosuppressive effects of cyclosporine A. Safety trade-offs between immune modulation and host defense; for instance, inhibiting the STING pathway necessitates careful consideration of infection risk. Furthermore, the dose-dependent effects of drugs, gender differences, and long-term safety require extensive clinical trials for validation. Research on cross-pathway interactions is vital, as the relationships between mitochondrial membrane permeabilization, mitophagy, and EV release are complex. Future multidisciplinary studies should clarify mechanisms, optimize drug design, and advance personalized treatment, ultimately enabling precision immune interventions.

## 5 Conclusion

This review elucidates that elevated circulating cf-mtDNA in blood serve not only as a indicator of cellular metabolic status, but also participate in the pathogenesis of various diseases. A critical observation is that mtDNA can activate multiple signaling pathways, such as the cGAS-STING pathway, inflammasomes, and TLR9, thereby initiating innate immune responses, RCD, and alterations in lipid metabolism. This perspective offers novel insights into the emergence and progression of specific clinical symptoms, while simultaneously presenting opportunities for the development of innovative therapeutic strategies. Although existing studies have examined the mechanisms of mtDNA release and the immune signaling pathways mediated by mtDNA, additional clarification of the key molecular complexities and refinement of experimental strategies remain necessary. Furthermore, future research should prioritize the development of mitochondrial-targeted pharmacological agents capable of regulating mitochondrial membrane integrity, mitigating oxidative stress, and sustaining mitochondrial autophagy functions, as well as inhibitors of PRR immune signaling pathways.
